# DNA interference-mediated screening of maternal factors in the chordate *Oikopleura dioica*

**DOI:** 10.1038/srep44226

**Published:** 2017-03-10

**Authors:** Tatsuya Omotezako, Masaki Matsuo, Takeshi A. Onuma, Hiroki Nishida

**Affiliations:** 1 Department of Biological Sciences, Graduate School of Science, Osaka University, 1-1Machikaneyama-cho, Toyonaka, Osaka 560-0043, Japan

## Abstract

The maternal contribution to the oocyte cytoplasm plays an important role during embryogenesis because it is involved in early cell fate specification and embryonic axis establishment. However, screening projects targeting maternal factors have only been conducted in a limited number of animal models, such as nematodes, fruit flies, and zebrafish, while few maternal genes have been analysed because of difficulties encountered in inhibiting gene products already expressed in the ovaries. Therefore, simple and efficient methods for large-scale maternal screening are necessary. The appendicularian *Oikopleura dioica* is a planktonic tunicate member of the chordates. Gonadal microinjection and a novel gene knockdown method, DNA interference (DNAi), have been developed for use in this animal with the aim of inhibiting gene functions during oogenesis within the gonad. In this study, we adapted these methods for large-scale maternal factor screening, and observed malformation phenotypes related to some maternal factors. Approximately 2000 (56.9%) ovary-enriched gene products were screened, of which the knockdown of seven encoding genes resulted in various abnormalities during embryonic development. Most of these were related to microtubules and cell adhesion-related proteins. We conclude that DNAi is a potentially powerful screening tool for the identification of novel maternal factors in chordates.

Maternal factors in this study refer to mRNAs or proteins that are loaded into the egg cytoplasm during oogenesis. They are fundamental resources that regulate developmental processes such as fertilisation, cell division, and embryonic axis specification before the start of zygotic expression^1^,^2^,^3^, as well as some early developmental processes after the maternal–zygotic transition. For example, maternal *postplasmic*/*PEM* RNAs show characteristic localisation patterns and play various roles in the regulation of embryonic axis specification and cell fate determination in ascidians^4^,^5^,^6^.

Although some maternal factors with important functions during embryogenesis have been predicted by conventional micromanipulation approaches such as deletion and cytoplasm transfer experiments using eggs, they have not yet been identified. For instance, vegetal hemisphere determinants are likely maternal proteins, but have thus far not been identified in any animals. As an example, the β-catenin protein is specifically translocated into the nuclei of vegetal cells in most invertebrates, resulting in the activation of vegetal cell fate-related genes^7^,^8^,^9^,^10^. β-catenin nuclear accumulation is regulated by an unknown vegetal determinant maternal protein localised within the oocyte^11^.

Functional screening is an effective means of detecting these unidentified genes. To inhibit the function of maternal factors, morpholino antisense oligonucleotides (MOs) are injected into oocytes, but this is not sufficient for two reasons. First, MOs injected into spawned eggs cannot inhibit the function of maternal proteins that have already been translated during oogenesis in the ovary. Second, the efficiency of injected MOs is unpredictable and varies depending on the target sequences. Therefore, reverse genetic screenings of maternal factors has not been carried out, except for *Caenorhabditis elegans* in which the ovary microinjection method is available^12^. On the other hand, forward genetics approaches to screen maternal factors have been applied to *C. elegans, Drosophila melanogaster*, and *Danio rerio*^1^,^2^,^3^,^13^,^14^. This technique involves the mating of animals for four generations to detect the maternal effects of mutant genes. However, because such screening takes considerable time and effort, only a limited number of genes have been screened to date, especially in chordates. Therefore, new experimental systems to identify novel maternal factors that regulate developmental processes during embryogenesis in chordates are required.

*Oikopleura dioica* is a planktonic tunicate belonging to the chordate phylum. It is used as a model organism in developmental biology because of a number of advantageous traits: (i) a small number of constituent cells and invariant cell lineages, (ii) a rapid development and short life cycle of 5 days, (iii) a transparent embryo and adult body, (iv) a compact genome of 70 Mb, and (v) publicly available genome browser and transcriptomic microarray data^15^,^16^. Recently, ovarian microinjection has been developed for use in this organism^17^. The *O. dioica* ovary has a multinuclear syncytium structure (a coenocyte containing many nurse and mitotic nuclei) that simultaneously forms 200–300 oocytes in half a day^18^. Injection of mRNA or DNA into the ovary during oogenesis results in these nucleic acids being inherited by multiple spawned eggs.

DNA interference (DNAi) is another advantageous method that has been devised for use in *O. dioica*. It involves the injection of PCR products to induce gene knockdown in a sequence-dependent manner^19^. This is a simple and inexpensive method for gene product screening because the preparation of PCR products is more straightforward than that of double strand RNA for RNA interference^17^. These techniques make *O. dioica* a suitable animal for analysing the function of maternal factors because their function can be inhibited during oogenesis^19^.

In the present study, we targeted maternal factors of *O. dioica* using DNAi, and show that this method is efficient for inhibiting the function of maternal factors. Approximately 2000 ovary-enriched genes were screened, and seven maternal factors were identified to induce various malformations of early embryos when inhibited. These factors encode proteins involved in cell cleavage, nuclear transportation, cell–cell adhesion, and DNA replication. *O. dioica* is therefore a useful experimental model for the analysis of maternal factor functions in chordates.

## Results

### Confirmation of the effect of DNAi on maternal genes

In a previous study, the efficiency of DNAi in the knockdown of maternal genes was indirectly shown for exogenously injected mRNA encoding a fluorescent protein^18^; however, there is no direct evidence for endogenous maternal genes. Therefore, first, we used DNAi to target the β-catenin protein, which is present in the oocytes of most animals, and mediates cell–cell adhesion in cooperation with cadherins^20^. It also regulates gene activation as a member of the canonical wnt pathway, resulting in the vegetal hemisphere specification in many invertebrates^7^,^8^,^9^,^10^,^20^.

To identify homologous proteins of β-catenin in *O. dioica*, tblastn was carried out querying the β-catenin protein sequence of the ascidian *Ciona intestinalis* (NP_001027779.1) using OikoBase^16^ (http://oikoarrays.biology.uiowa.edu/Oiko/index.html). Potential candidates were analysed to generate a phylogenetic tree. Among the maternally expressed genes, two homologues (GSOIDP00011813001 and GSOIDG00004053001 in Oikobase) were identified and named β-catenin1 and β-catenin2, respectively.

**Figure 1:**
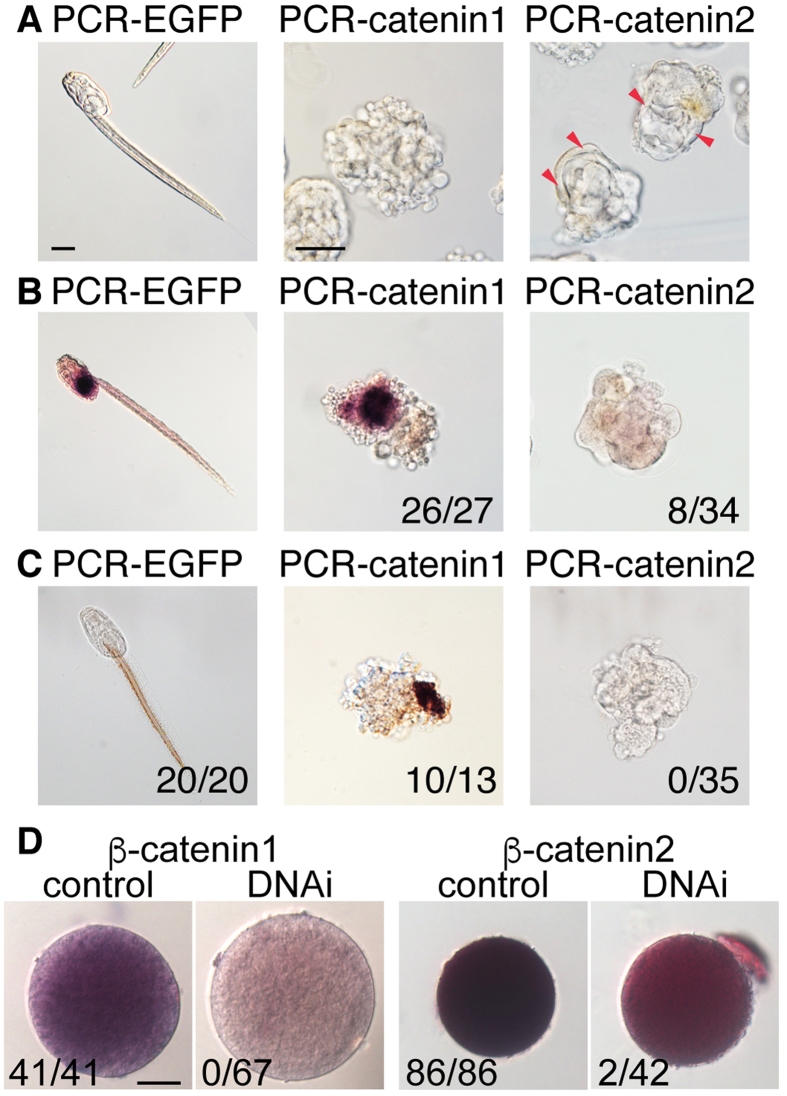
DNAi-mediated knockdown of β-catenin homologues and developmental phenotypes. (**A**) Morphology of tadpole larvae injected with PCR-EGFP (control), PCR-catenin1 or PCR-catenin2 at 7 hpf. Scale bar, 50 μm. Red arrowheads show an epidermis-like structure. (**B**) Histochemical staining for the endoderm differentiation marker alkaline phosphatase (ALP) in larvae injected with PCR-EGFP, PCR-catenin1 or PCR-catenin2. Total numbers of larvae with ALP signals are shown at the bottom. (**C**) Histochemical staining for the muscle differentiation marker acetylcholinesterase (AChE) in larvae injected with PCR-EGFP, PCR-catenin1 or PCR-catenin2. Total numbers of larvae with AChE staining are shown at the bottom. (**D**) Whole-mount *in situ* hybridization for β-catenin1 and β-catenin2 in oocytes injected with PCR-Kaede as control, PCR-catenin1 or PCR-catenin2. Total numbers of oocytes showing each targeted mRNA signal at a normal level are shown at the bottom. Scale bar, 50 μm.

PCR amplification products (PCR-catenin1 and PCR-catenin2) covering the partial sequences of β-catenin1 and β-catenin2 (545–1042 and 537–1066 nucleotides counted from the methionine start codon, respectively) were injected into the *O. dioica* ovary. Following the injection of PCR-catenin1, cell adhesion was reduced at the 32-cell stage, and embryonic cells became dissociated after the hatching stage (Fig. 1A). These results indicate that PCR-catenin1 affects cell–cell adhesion. In contrast, PCR-catenin2-injected embryos developed into a mass of cells with an epidermis-like structure at the periphery at 7 h post-fertilisation (hpf) (Fig. 1A, red arrowheads).

To investigate the effect on endoderm differentiation, we performed histochemical staining for the endoderm marker alkaline phosphatase (ALP)^8^,^21^. The injection of PCR-catenin2, but not PCR-catenin1, resulted in loss of ALP activity, indicating that PCR-catenin2 suppressed endoderm specification (Fig. 1B). To examine DNAi effects on differentiation of another tissue cells, acetylcholinesterase (AchE) activity that is known as a muscle marker^22^ was investigated. In PCR-catenin1 injected animals, muscle differentiation was observed. On the other hand, injection of PCR-catenin2 resulted in loss of muscle differentiation (Fig. 1C). Improper specification of the vegetal hemisphere caused by β-catenin2 inhibition could also inhibit muscle differentiation. On the other hand, epidermis-like epithelial structure was observed in β-catenin2 injected animal (Fig. 1A, arrowhead). Therefore, the absence of endoderm and muscle differentiation was specifically promoted by knockdown of catenin-2, but not by nonspecific toxic effect. Furthermore, whole-mount *in situ* hybridization revealed decreases of *β-catenin1* and *β-catenin2* mRNA contents in oocytes that were injected with PCR-catenin1 or PCR-catenin2, respectively (Fig. 1D), suggesting that the obtained phenotypes were induced by reduction of maternal products of the targeted genes. It is likely that β-catenin1 and β-catenin2 mediate cell adhesion and the wnt signaling cascade, respectively, in *O. dioica*. Similar results were obtained following the inhibition of β-catenin in other organisms, especially in ascidians^20^, revealing that DNAi can effectively inhibit the functions of endogenous maternal genes. Of particular note is that the two distinct functions of β-catenin of most animals are separated into two β-catenin homologues in *O. dioica*, while most organisms only contain one β-catenin protein^20^.

### Construction of a female-specific cDNA library

For efficient maternal factor screening, an adult female-specific cDNA library was produced by subtraction cloning between female cDNAs (obtained from the trunk and ovary of maturing adult females) and male cDNAs (obtained from the trunk and testis of maturing adult males). During the subtraction process, normalisation of the abundance of different cDNAs in the library was simultaneously carried out to equalise the prevalence of each cDNA using the PCR-Select™ cDNA Subtraction Kit (Clontech).

**Figure 2:**
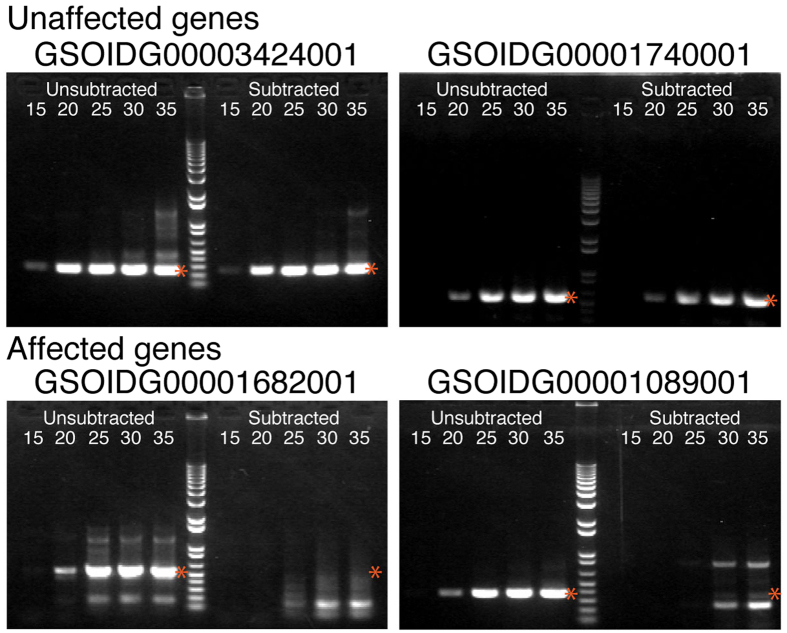
Evaluation of subtraction for ovary-enriched cDNAs. cDNAs corresponding to four genes, GSOIDG00003424001, GSOIDG0000174001, GSOIDG00001682001, and GSOIDG00001089001, were amplified by PCR to determine their copy numbers in female-specific (Subtracted) and Unsubtracted libraries. Gel images show products of PCR amplification of each cDNA at 15, 20, 25, 30, and 35 cycles. The amplification products of female-specific cDNAs were expected to be visible at the same number of cycles for both libraries, while the amplification of subtracted cDNAs was expected to require more cycles to become visible. The red asterisk shows the expected position of each amplified fragment. Fragments of other lengths would be nonspecific. See also Fig. S1 for the results of other genes.

To evaluate subtraction of the library, we compared the amount of cDNAs specifically expressed in females with those expressed in both females and males by PCR (Fig. 2, Fig. S1). The amount of cDNA corresponding to the following seven mRNAs was compared between the subtracted library and non-subtracted library: GSOIDG00003424001 (expressed only in sexually maturing females based on Oikobase microarray data), GSOIDG00001740001 (expressed 11-fold higher in females than in males), GSOIDG00001682001 (expressed 2.1-fold in females), GSOIDG00001134001 (expressed 1.7-fold in females), GSOIDG00001205001 (expressed less in females than in males), GSOIDG00001089001 (expressed less in females than in males), and GSOIDG00001739001 (expressed less in females than in males).

The non-subtracted library was created using the same process as the subtracted library, except for the subtraction step of male cDNAs. Female-specific cDNAs were expected to be amplified at the same level in both subtracted and non-subtracted libraries, while the amount of cDNAs expressed both in females and males in the subtracted library was expected to be lower than in the non-subtracted library. GSOIDG00003424001 and GSOIDG00001740001 were amplified to the same extent in both libraries (upper two panels in Fig. 2). In contrast, GSOIDG00001134001, GSOIDG00001205001, GSOIDG00001739001 (Fig. S1) and GSOIDG00001089001 (lower two panels in Fig. 2) were successfully subtracted because they required more rounds of amplification in the subtracted library to produce faintly visible bands in the gels. These results indicate that cDNAs corresponding to mRNAs expressed 11-fold higher in females than males were not subtracted, while cDNAs corresponding to mRNAs expressed 2-fold higher in females than males were successfully reduced by the subtraction step.

These results enabled the number of cDNA species contained in the subtracted library to be estimated. Investigation of microarray expression data in Oikobase showed that maturing females (trunk + ovary) express 3564 of a total of 16748 genes at least 2-fold higher than males (trunk + testis). Therefore, the library is estimated to contain cDNA species corresponding to fewer than 3564 genes.

### Optimisation of mixing PCR products

To increase the DNAi screening efficiency, we used a mixture of PCR products targeting several distinct genes. To optimise the number of clones for the mixture, the knockdown efficiency of mixtures of PCR products targeting five or 10 genes was compared. In this experiment, one PCR product targeting the Brachyury gene (PCR-Bra), which promotes notochord malformation^17^, and four or nine other genes that induce no malformation were mixed. The final concentration of each PCR product was adjusted to approximately 0.2 μg/μL.

**Figure 3:**
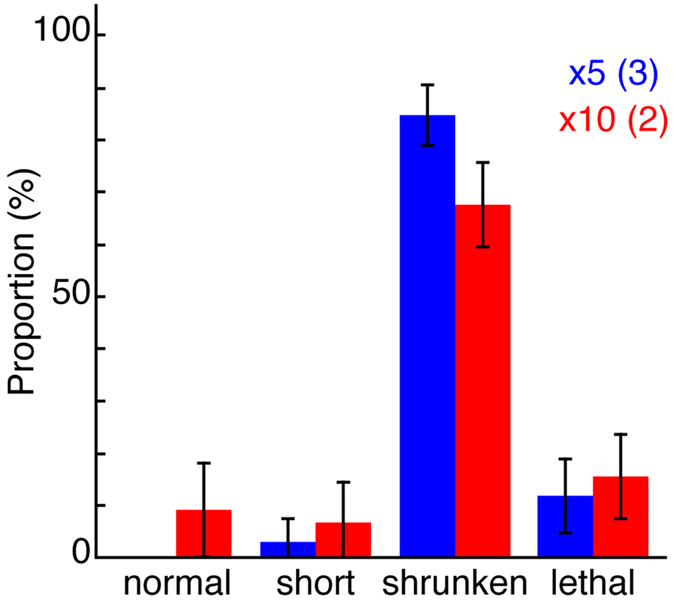
Evaluation of pooling PCR products for the knockdown of multiple genes. Proportions of each of the phenotypes of hatched larvae after the injection of PCR-Bra plus PCR products targeting four (×5, blue) or nine other genes (×10, red). Each PCR product was diluted in the pool at approximately 0.2 μg/μL. Numbers in parentheses represent those of independent injection experiments. Bars, standard deviation.

The PCR mixture of five products induced a morphological shrunken tail phenotype in 85 ± 6% (mean ± standard deviation) of hutch larvae, while none showed normal tail elongation (Fig. 3). In comparison, the PCR mixture of 10 products induced the shrunken tail phenotype in 68 ± 8%, while 9 ± 9% showed normal tail elongation (Fig. 3). Because the mixture of 10 products reduced the efficiency of PCR-Bra, the mixture of five PCR products was used for the following screening project.

### Injection of pools of PCR products

**Figure 4:**
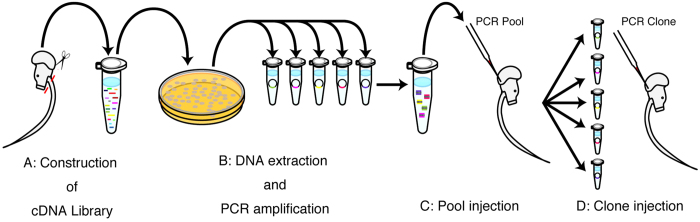
Schematic of the screening process. (**A**) The female cDNA-enriched library was constructed by subtraction cloning. Tails were removed prior to RNA isolation. (**B**) Colonies on agar plates were randomly picked and the plasmid inserts were amplified by PCR. (**C**) A pool of five PCR products was injected into the ovary. (**D**) When abnormal embryogenesis of spawned eggs occurred after injection of the PCR pool, each PCR product was separately injected to identify the clone responsible for the original malformation.

To identify maternal genes that affect the developmental processes of spawned eggs, pools of five PCR products were introduced into sexually maturing female gonads. Figure 4 shows the screening scheme. We manually removed the tails of females and males and constructed a subtracted library in which female cDNAs were enriched (Fig. 4A). *Escherichia coli* clones including female-enriched cDNAs were picked randomly and plasmid inserts were amplified by PCR in separate tubes (Fig. 4B). Then, five PCR products were pooled and injected into ovaries to identify candidates that induce malformations (Fig. 4C). If a certain pool induced a malformation, each of the five PCR products was separately injected into an ovary to identify the clone responsible for inducing the malformation (Fig. 4D).

To evaluate the knockdown effect of PCR products, injected females were kept overnight at 20 °C until they spawned eggs. Eggs were then fertilised and cultured until the hatched larval stage (3 hpf). The effects of knockdown were evaluated by observing morphological abnormalities of hatched larvae at 3 hpf for pools and 3, 5, and 7 hpf for identified clones. PCR products responsible for causing malformations in more than 90% of injected larvae (i.e., larvae with H2B-mCherry fluorescence as the injection marker) were considered candidates for producing a maternal effect and were used for further analysis. PCR products promoting malformations with a lower penetrance were excluded from the present analysis. A total of 600 pools (3000 clones) were screened, of which 26 pools showed malformations in the hatched larvae (3 hpf; Fig. S2). These pools were regarded as candidate pools containing clones that affect developmental processes.

### Isolation of responsible cDNA clones

**Figure 5:**
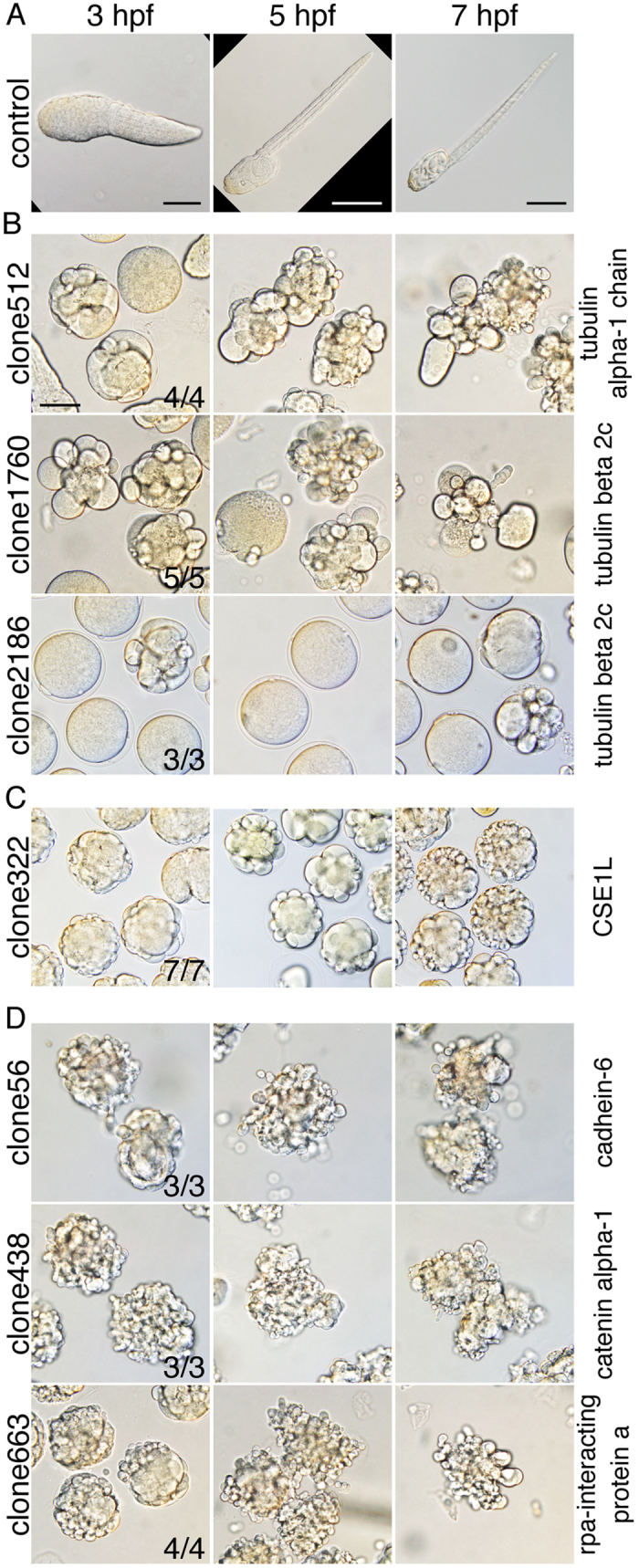
Phenotypes of hatched larvae injected with the seven PCR clones at 3, 5, and 7 hpf. Clone numbers are indicated on the left of the panels, and gene product names are shown on the right. (**A**) Normal noninjected larvae at 3, 5, and 7 hpf. Observed abnormalities were categorized as cell division arrest (**B**), mid-embryogenesis morphogenesis arrest (**C**), and cell dissociation (**D**). Total number of batches that showed the malformation phenotypes in more than 90% of injected larvae is shown at the bottom right. Scale bar, 50 μm.

To identify the cDNA clones responsible for inducing malformations, PCR products from the 26 pools (130 clones) were injected separately. In seven pools, a single clone was successfully identified to reproduce the malformation (Fig. 5, Table S1), while injection of the other four clones resulted in normal development. None of the five clones from the remaining 19 pools reproduced the original malformation, suggesting that the original malformation observed in larvae injected with these pools resulted from a low quality of eggs. This type of aberrant embryogenesis was occasionally observed even in un-manipulated animals. Approximately, 5% of un-manipulated matured female spawns oocytes of low quality.

To further characterise the phenotype of the seven identified clones, larvae were observed at 3, 5, and 7 hpf. In *O. dioica* development, the animal hatches at 3 hpf, rudimentary trunk organs such as the nervous system and digestive organs become visible at 5 hpf, and morphogenesis is almost complete at 7 hpf (Fig. 5A). The phenotypes of the seven clones could be categorised into three types. First, injection of clones 512, 1760, and 2186 affected cell cleavage (Fig. 5B), and thus eggs did not divide or embryogenesis was arrested after a few abnormal cell cleavages. In these embryos, the presence of multiple nucleus-like structures within a single cell was visualised by the red fluorescence of Histone 2B:mCherry (Fig. S3A), of which mRNA had been coinjected as an injection marker (see Methods section). Sequencing of clones 512, 1760, and 2186 revealed that they encode *tubulin alpha-1* and two similar but distinct *tubulin beta 2c* genes, respectively, according to Oikobase gene annotation. BLAST search on the NCBI website also confirmed that the clones encode tubulins, which seems reasonable because tubulins mediate cell division.

Second, injection of clone 322 resulted in developmental arrest at mid-embryogenesis (Fig. 5C). Cell division appeared normal during the early embryonic stages; however, the embryos failed to undergo normal morphogenesis and developed into a disorganised cell mass. Sequence analysis showed that the clone encodes chromosome segregation 1-like (CSE1L) protein. CSE1L exports importin-α proteins from inside the nucleus to the cytoplasm for recycling^23^. The phenotype is therefore likely to be caused by insufficient general nuclear transportation.

Third, injection of clones 56, 438, and 663 resulted in cell dissociation, and the embryos developed into a mass of tiny cells (Fig. 5D). In animals injected with PCR products of clones 56 and 438, cell adhesion was reduced as early as the 16-cell or 32-cell stage (Fig. S3B). In contrast, in embryos injected with clone 663, cell adhesion appeared normal during the early embryonic stage (Fig. S3), but cells had gradually dissociated by hatching. Sequence analysis showed that clones 56, 438, and 663 encode the cadherin-6 precursor, catenin alpha-1, and replication protein A (rpa)-interacting protein a, respectively. The observed phenotypes resulting from the injection of clones 56 and 438 were reasonable because cadherin and catenin alpha are components of the adherence junction complex. Similarly, cell dissociation induced by clone 663 could be caused by abnormal cell proliferation and cell death resulting from defects in DNA replication, repair, and recombination processes that are known to involve rpa-interacting protein a^24^.

To confirm that phenotypes of the seven clones were promoted by knockdown of the targeted genes, two PCR products encoding distinct and non-overlapping sequences were injected for each gene (Table S2). These pair of PCR products reproduced the same phenotype as those observed in screening process except for clone 56. In several genes, penetrance of the phenotype was less than 90% of larvae. But feature of the phenotype was reproduced even in these cases and these results supported DNAi specificity.

To confirm that these phenotypes were promoted by reduction of the maternal gene products, whole-mount *in situ* hybridization was carried out using unfertilized eggs. Reduction of the amount of targeted mRNAs was observed in oocytes injected with PCR products targeting for clones 56, 322, 438, 512, 1760, and 2186 (Fig. S4). However, reduction was not evident in clone 663 (data not shown). Therefore, the effects on rpa-interacting protein a could be zygotic. Taking these results together with those of β-catenin (Fig. 1D), DNAi is effective in reduction of maternal gene products in most cases.

## Discussion

In the present study, we carried out cDNA screening of maternal factors in the chordate *O. dioica*. The effects of DNAi on maternal factors were first assessed by microinjecting ovaries with PCR products encoding β-catenin. This technique was shown to be sufficiently potent to inhibit the functions of maternal mRNAs, and possibly maternal proteins whose functions could not be inhibited by the microinjection of MO or dsRNA into spawned eggs.

We screened a total of 3000 clones in the ovary-enriched cDNA library to analyse the functions of maternal factors. Because clones were picked up at random, cDNAs corresponding to an identical gene were picked multiple times. Taking this overlap into account, the microinjection of PCR products corresponded to inhibiting the function of 2028 genes (Fig. S5). Because our subtracted and normalised cDNA library is estimated to contain 3564 genes, this covers approximately 57% of genes in the library. However, as shown in Table S2 penetrance of DNAi phenotypes varied among injected PCR products. Efficiency of DNAi might depend on target sequences. The larger region target sequence covers, the greater chance one would see the phenotype with. Since only clones that promoted malformation in more than 90% of injected embryos were considered as candidate genes in the present study, it seems that some genes were missed even though they are crucial for embryogenesis.

Seven genes were demonstrated to have functions in embryonic development, encoding tubulins, CSE1L, cadherin, catenin alpha, and rpa-interacting protein a. These proteins play roles in cell cleavage, nuclear transportation, cell–cell adhesion, and DNA replication. Two pieces of evidence suggest that the obtained phenotypes result from maternal effects rather than the suppression of zygotic gene expression after fertilization. First, the expression pattern of clones 322, 1760, and 2186 strictly follows that of maternal genes in which mRNA levels gradually decrease during embryonic development prior to hatching, probably without zygotic transcription according to Oikobase microarray data (Fig. S6). Second, the inhibition of clones 512, 1760, 2186 (encoding tubulins), 56 (encoding cadherin), and 438 (encoding catenin alpha) showed abnormal phenotypes as early as the cleavage stage, possibly before the start of zygotic expression. However, it is still possible that the observed phenotypes were caused by knockdown of the zygotic transcripts rather than the maternal ones in several clones.

The prevalence of genes that induce aberrant phenotypes in the maternal screening of *O. dioica* was lower than that previously identified for other animals. These differences might be caused by technical differences of gene inhibition method rather than by difference of gene functions among animals. For example, in *C. elegans*, RNAi screening for maternal genes showed that 81 out of 350 clones induced malformations that were mainly abnormalities of oogenesis^12^. However, no oogenesis malformations were observed in *O. dioica*. This difference might reflect variations in microinjection timing. For example, the injection of dsDNA into the ovary of a Day 4 animal (half a day before spawning at 20 °C) could be too late to interfere with oogenesis because sufficient protein might have already accumulated for the completion of oogenesis. Similarly, the timing of ovarian injection is also important for the knockdown of maternal products required for embryogenesis after spawning. In fact, in the case of CSE1 like, injection into animals during the late maturing process (when the oocyte outline is faintly visible under a microscope) was shown to be too late to achieve developmental arrest in the present study. This indicates that it is important to inject animals as early as possible during oogenesis.

In the present screening, we used a subtracted cDNA library lacking housekeeping genes, which may explain why the occurrence of genes inducing aberrant phenotypes was so low. We compared the expression of the seven clones using Oikobase microarray data. Cadherin-6 (clone 56), CSE1L (clone 322), and catenin alpha-1 (clone 438) were expressed in the ovary but not the testis. One tubulin beta 2c clone (clone 2186) was expressed 8.6 times while another tubulin beta 2c clone (clone 1760) was expressed 1.4 times higher in the ovary than the testis. Data were not available for catenin alpha-1 (clone 512). Although these types of protein appear to be required for cellular housekeeping activities, they are nevertheless enriched in the gonads.

DNAi was previously estimated to reduce the amount of target mRNAs by 70–80%, while protein synthesis from exogenously injected mRNA encoding fluorescent proteins was decreased by 25–60%^19^. Therefore, it is possible that many maternal factors were undetected by the present screening, although it is not possible to quantify this. Additionally, we used pools of five PCR products for the first step of screening. Although we confirmed that such a pool resulted in sufficient functional inhibition at the beginning, we observed that the injection of a single clone in the second step of screening produced a greater effect than the injection of pooled products (Fig. 5 and S2). For example, the injection of clones 2186–2190 only arrested cell division after several rounds, while the injection of single clone 2186 arrested cell division at the first cleavage in most cases. A similar phenomenon was observed for clones 512 and 1760. Therefore, pool injections may produce a weaker effect than clone injections even though PCR product concentrations are the same (0.15–0.20 μg/μL). It is conceivable that each PCR product in a pool might compete for machinery that mediates DNAi.

Despite these difficulties, *O. dioica* remains a useful model for analysing and screening maternal genes. Further studies using this technique are expected to identify the function of additional maternal genes.

## Methods

### Laboratory culture of *O. dioica*

Wild *O. dioica* were previously collected from Sakoshi Bay and Tossaki Port in Hyogo prefecture, Japan, and have been cultured for more than 2 years in the laboratory^15^,^17^,^19^. Histochemical detection of alkaline phosphatase^8^,^21^ and acetylcholinesterase^19^ were carried out as previously described.

### cDNA library construction of ovary-enriched transcripts

Total RNA was prepared from seven pre-matured female animals in which the oocyte outline was not yet visible in the ovary under a stereomicroscope, and eight pre-matured male animals in which the testis was becoming brown in colour. This stage corresponds to animals that will spawn in approximately 8–12 h, and is the stage when microinjection was performed. Tails of the animals were removed with a knife and the trunk and ovary were used. Total RNA was extracted using the Nucleospin RNA XS (MACHEREY-NAGEL, Düren, Germany) and reverse-transcribed using the SMARTer™ PCR cDNA Synthesis Kit (Clontech, California, USA) according to the manufacturer’s instructions. cDNAs were amplified by PCR for 14 cycles for females and 12 cycles for males. Subtraction and normalisation were carried out using the PCR-Select^™^ cDNA Subtraction Kit (Clontech) according to the manufacturer’s instructions.

### Subtracted library quality check

To evaluate the subtraction efficiency, the amount of cDNA for ovary-specific genes (GSOIDG00003424001 and GSOIDG00001740001 in Oikobase) and ovary non-specific genes (GSOIDG00001682001, GSOIDG00001134001, GSOIDG00001205001, GSOIDG00001089001, and GSOIDG00001739001) was estimated by PCR and compared between subtracted and nonsubtracted libraries. These genes were selected based on Oikobase microarray data (http://oikoarrays.biology.uiowa.edu/Oiko/expression_matrix.html). PCR conditions were: 95 °C for 30 s, 55 °C for 30 s, and 72 °C for 1 min. The amplification used gene-specific primers, and PCR products were run on gel electrophoresis after 15, 20, 25, 30, and 35 cycles.

### Preparation of PCR products for microinjection

dsDNAs in the library were subcloned into a vector using TOPO^®^ TA cloning for sequencing (ThermoFisher, Waltham, Massachusetts, USA), and were then transformed into DH5α *Escherichia coli*. Each colony was randomly picked, and underwent plasmid extraction and purification using the PureYield^™^ Plasmid Miniprep System (Promega, Madison, Wisconsin, USA). PCR products for microinjection were amplified using KOD plus (TOYOBO, Osaka, Japan) using the T3 primer and T7 primer. Products were purified by phenol–chloroform and ethanol precipitation, and dissolved in 30 μL of water. For a pooled mixture of five PCR products, all products were amplified separately using five plasmid templates and then mixed into a tube for purification. This PCR mixture was also diluted into 30 μL of water after ethanol precipitation, such that the DNA concentration of each gene was approximately 0.2 μg/μL.

### Microinjection into the ovary

Ovarian microinjection was performed as previously described^19^. Injected DNA is incorporated into multiple oocytes during oogenesis because each pro-oocyte is connected to a shared cytoplasm through pores known as the ring canal^18^. Injected DNA diffuses into the ovary along a concentration gradient, and is incorporated into 20–30% of spawned eggs. Therefore, mRNA encoding H2B-EGFP or H2B- mCherry was co-injected with PCR products as an injection marker, and only eggs demonstrating EGFP or mCherry fluorescence were used for analysis, as described previously^17^. The final concentration of each injected PCR product was approximately 0.20 μg/μL after the addition of phenol red solution (10 mg/ml). DNA was injected into the maturing ovary after it had expanded to fill the entire posterior region of the animal but before it had reached its final thickness and the oocyte boundary was visible. Injected animals spawned eggs approximately 12 h after microinjection, and these eggs were then artificially fertilised by sperm.

### Whole-mount *in situ* hybridization

To evaluate knockdown effects on maternal mRNAs, whole-mount *in situ* hybridization^17^,^22^,^25^ was carried out. RNA probes were designed for the same sequences to PCR products used for DNAi. Eggs were treated with 0.05% Actinase E (Funakoshi, Tokyo, Japan) and 1% sodium thioglycolate (Sigma, Tokyo, Japan) in seawater for 4 min at room temperature to digest the vitelline membrane, and fixed with 4% paraformaldehyde in 0.1 M MOPS and 0.5 M NaCl. To remove injected PCR products, specimens were treated with 2 U/ml Turbo DNase and 20 μg/ml proteinase K (Thermo Fisher Scientific, Yokohama, Japan) at 37 °C for 15 min, fixed again with 4% paraformaldehyde, and subjected for hybridization with DIG-labeled RNA probes in hybridization buffer (50% de-ionized formamide, 5X SSC, 0.1% Tween-20, 50 mg/ml Heparin, 100 mg/ml *E. coli* tRNA) at 60 °C for 4 hour. After hybridization, probes were washed out, and specimens were incubated with anti-DIG-AP antibody (Roche). Signal detection was carried out as described previously^17^.

## Additional Information

**How to cite this article**: Omotezako, T. *et al*. DNA interference-mediated screening of maternal factors in the chordate *Oikopleura dioica. Sci. Rep.* **7**, 44226; doi: 10.1038/srep44226 (2017).

**Publisher's note:** Springer Nature remains neutral with regard to jurisdictional claims in published maps and institutional affiliations.

## Supplementary Material

Supplemental Figure

## References

[b1] Schüpbach T. & Wieschaus E. Female sterile mutations on the second chromosome of *Drosophila melanogaster*. I. maternal effect mutations. Genetics 121, 101–117 (1989). 2492966 10.1093/genetics/121.1.101 PMC1203592

[b2] Hekimi S., Boutis P. & Lakowski B. Viable maternal-effect mutations that affect the development of the nematode *Caenorhabditis elegans*. Genetics 141, 1351–1364 (1995). 8601479 10.1093/genetics/141.4.1351 PMC1206872

[b3] Dosch R. . Maternal control of vertebrate development before the midblastula transition: mutants from the zebrafish I. Dev. Cell 6, 771–780 (2004). 15177026 10.1016/j.devcel.2004.05.002

[b4] Makabe K. W. . Large-scale cDNA analysis of the maternal genetic information in the egg of Halocynthia rorezi for a gene expression catalog of ascidian development. Development 128, 2555–2567 (2001). 11493572 10.1242/dev.128.13.2555

[b5] Prodon F., Yamada L., Shirae-Kurabayashi M., Nakamura Y. & Sasakura Y. Postplasmic/PEM RNAs: a class of localized maternal mRNAs with multiple roles in cell polarity and development in ascidian embryos. Dev. Dyn. 236, 1698–1715 (2007). 17366574 10.1002/dvdy.21109

[b6] Makabe K. & Nishida H. Cytoplasmic localization and reorganization in ascidian eggs: Role of *postplasmic*/*PEM* RNAs in axis formation and fate determination. WIREs Dev. Biol. 1, 501–518 (2012). 10.1002/wdev.54 23801532

[b7] Darras S., Gerhart J., Terasaki M., Kirschner M. & Lowe C. J. β-catenin specifies the endomesoderm and defines the posterior organizer of the hemichordate *Saccoglossus kowalevskii*. Development 138, 959–970 (2011). 21303849 10.1242/dev.059493 PMC3035098

[b8] Imai K., Takeda N., Satoh N. & Satou Y. β-catenin mediates the specification of endoderm cells in ascidian embryos. Development 127, 3009–3020 (2000). 10862739 10.1242/dev.127.14.3009

[b9] Logan C. Y., Miller J. R., Ferkowicz M. J. & McClar D. R. Nuclear beta-catenin is required to specify vegetal cell fates in the sea urchin embryo. Development 126, 345–357 (1999). 9847248 10.1242/dev.126.2.345

[b10] Wikramanayake A. H. . An ancient role for nuclear beta-catenin in the evolution of axial polarity and germ layer segregation. Nature 426, 446–450 (2003). 14647383 10.1038/nature02113

[b11] Nishida. H. Localized regions of egg cytoplasm that promote expression of endoderm-specific alkaline phosphatase in embryos of the ascidian *Halocynthia roretzi*. Development 118, 1–7 (1993).

[b12] Piano F., Schetter A. J., Mangone M., Stein L. & Kemphues K. J. RNAi analysis of genes expressed in the ovary of *Caenorhabditis elegans. Curr. Biol*. 10, 1619–1622 (2000). 10.1016/s0960-9822(00)00869-1 11137018

[b13] Kemphues K. J., Kusch M. & Wolf N. Maternal-effect lethal mutations on linkage group II of *Caenorhabditis elegans*. Genetics 120, 977–986 (1988). 3224814 10.1093/genetics/120.4.977 PMC1203589

[b14] Kishimoto Y., Koshida S., Furutani-Seiki M. & Kondoh H. Zebrafish maternal-effect mutations causing cytokinesis defect without affecting mitosis or equatorial vasa deposition. Mech. Dev. 121, 79–89 (2004). 14706702 10.1016/j.mod.2003.10.001

[b15] Nishida H. Development of the appendicularian *Oikopleura dioica*: Culture, genome, and cell lineages. Dev. Growth. Differ. 50, 239–256 (2008). 10.1111/j.1440-169X.2008.01035.x 18494706

[b16] Danks. G. . OikoBase: a genomics and developmental transcriptomics resource for the urochordate *Oikopleura dioica*. Nucleic Acids Res. 41, D845–D853 (2013). 23185044 10.1093/nar/gks1159 PMC3531137

[b17] Omotezako T., Nishino A., Onuma T. A. & Nihida H. RNA interference in the appendicularian *Oikopleura dioica* reveals the function of Brachyury gene. Dev. Genes. Evol. 223, 261–267 (2013). 23494664 10.1007/s00427-013-0438-8

[b18] Ganot P., Bouquet J. M., Kallsøe T. & Thompson E. M. The *Oikopleura* coenocyst, a unique chordate germ cell permitting rapid, extensive modulation of oocyte production. Dev. Biol. 302, 591–600 (2007). 17126826 10.1016/j.ydbio.2006.10.021

[b19] Omotezako T., Onuma T. A. & Nishida H. DNA interference: DNA-induced gene silencing in the appendicularian *Oikopleura dioica*. Proc. Biol. Sci. 282, 20150435 (2015). 25904672 10.1098/rspb.2015.0435 PMC4424657

[b20] McCrea P. D. & Gu D. The catenin family at a glance. J. Cell Sci. 123, 637–642 (2010). 20164302 10.1242/jcs.039842 PMC2823574

[b21] Kawai N., Iida Y., Kumano G. & Nishida H. Nuclear accumulation of β-catenin and Transcription of Downstream Genes Are Regulated by Zygotic Wntα and Maternal Dsh in Ascidian Embryos. Dev. Dyn. 236, 1570–1582 (2007). 17474118 10.1002/dvdy.21169

[b22] Nishino A., Satou Y., Morisawa M. & Satoh N. Muscle actin genes and muscle cells in the appendicularian, *Oikopleura longicauda*: phylogenetic relationships among muscle tissues in the urochordates. J. Exp. Zool. 288, 135–150 (2000). 10931497

[b23] Hood J. K. & Silver P. A. Cse1p is required for export of Srp1p/importin-α from the nucleus in *Saccharomyces cerevisiae*. J. Biol Chem. 273, 35142–35146 (1998). 9857050 10.1074/jbc.273.52.35142

[b24] Jullien D., Görlich D., Laemmli U. K. & Adachi Y. Nuclear import of RPA in Xenopus egg extracts requires a novel protein XRIPalpha but not importin alpha. EMBO J. 18, 4348–4358 (1999). 10428972 10.1093/emboj/18.15.4348 PMC1171510

[b25] Takatori N., Kumano G., Saiga H. & Nishida H. Segregation of germ layer fates by nuclear migration-dependent localization of Not mRNA. Dev. Cell 19, 589–598 (2010). 20951349 10.1016/j.devcel.2010.09.003

