# Supplementary material for: DNA interference-mediated screening of maternal factors in the chordate Oikopleura dioica
Source: Sci Rep. 2017 Mar 10;7:44226. doi: 10.1038/srep44226 (PMC5345011; doi:10.1038/srep44226)
Supplement: Supplemental Figure [file srep44226-s1.pdf]

SuppREmentary Information for Publication

DNA interference-mediated screening of maternal factors in the chordate Oikopleura dioica

Tatsuya Omotezako*, Masaki Matsuo, Takeshi A. Onuma & Hiroki Nishida*

Department of Biological Sciences, Graduate School of Science, Osaka University

Fig. S1. Evaluation of the achievement of subtraction. cDNAs corresponding to three genes, GSOIDG00001134001, GSOIDG0001205001, GSOIDG00001739001, were amplified by PCR determine their copy numbers in female-specific (Subtracted) and Unsubtracted libraries in the same manner as Fig. 2. The red asterisk shows the expected position of each amplified fragment. Fragments of other lengths would be nonspecific.

Fig. S2. Phenotypes of hatched larvae injected with 26 PCR pools, which promoted malformation at 3 hpf. Clone 11–15 represents a pool of five clones. Scale bar, 50 μm.

Fig. S3. Knockdown phenotypes of tubulin and cell adhesion molecule. (A) Embryos injected with DNA of clone 512, which encodes tubulin alpha-1. Multiple nucleus-like structures are present within an undivided single cell. Nuclei were visualized by red fluorescence of Histone 2B:mCherry. (B) Effects of clones 56 (cadherin-6 precursor), 438 (catenin alpha-1), and 663 (rpa-interacting protein a) were observed at the 32-cell stage. Cell adhesion was reduced and compaction of blastomeres was incomplete in embryos injected with clones 56 and 438. Red arrowheads show embryos that inherited the injection marker and show the phenotype. In contrast, this phenotype was not evident in embryos injected with clone 663. Blue arrowheads indicate embryos that inherited injection marker but show normal cell–cell adhesion. Embryos with no arrowhead did not inherit the injection marker (EGFP fluorescence was not observed). Scale bar, 50 μm.

Fig. S4. DNAi decreased amounts of the maternal mRNAs. Expression of each gene targeted by DNAi was detected in unfertilized eggs by whole-mount in situ hybridization. Eggs that were injected with Kaede PCR product were regarded as control. Total numbers of oocytes showing signal at a normal level are shown at the bottom. Scale bar, 50 μm.

Fig. S5. Calculated relationship between the number of screened clones and gene number. Since each clone is randomly picked from the normalised cDNA library, the number of inhibited genes gradually saturates as the number of screened clones increases. Injecting PCR products of 3000 clones correspond to inhibiting the functions of 2028 genes.

Fig. S6. Gene expression pattern of each clone. The temporal expression pattern of each clone was plotted using Oikobase microarray data. The ordinate represents relative expression compared with that in the oocyte. Clones 512 and 663 were not registered in Oikobase microarray data.

Table S1. Genes of responsible clones. Clone No., protein name of the candidate genes, Gene ID in Oikobase, DNAi target position and length of PCR products of the clones are shown. Target position is indicated as base position from initiating methionine in the cDNA.

Table S2. Target specificity of DNAi. PCR products which covers two distinct regions of each cDNA without any overlap were injected. Targeting position (upper column) and ratio of malformation in injected larvae (lower column) are shown.

Fig. S1

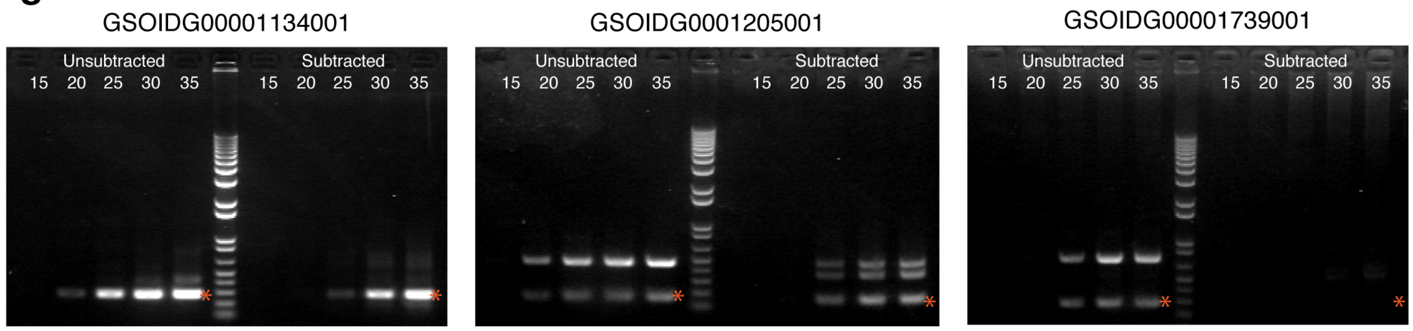
GSOIDG00001134001
GSOIDG0001205001
GSOIDG00001739001
Unsubtracted
Subtracted
15 20 25 30 35
15 20 25 30 35

Fig. S2

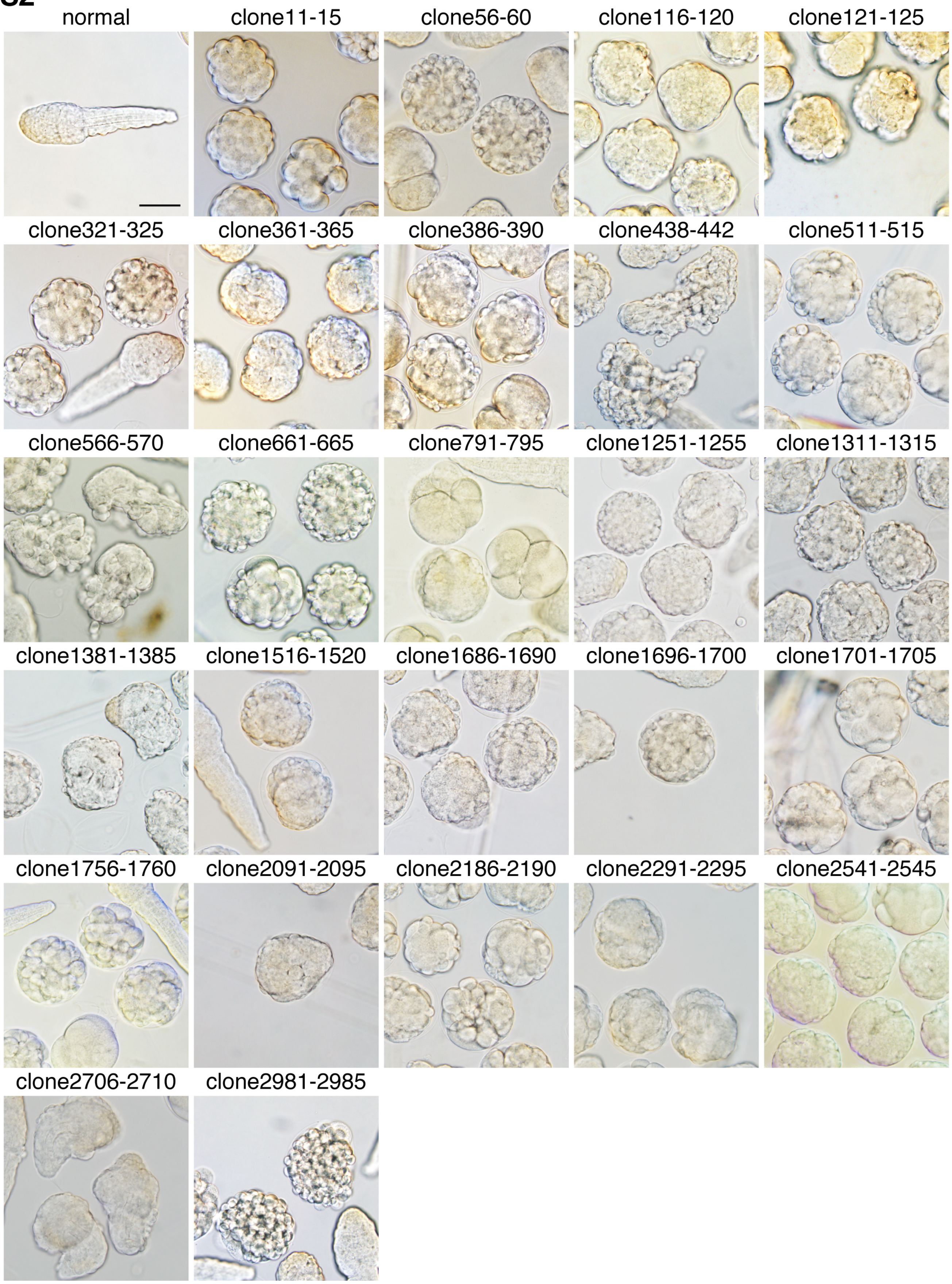
normal
clone11-15
clone56-60
clone116-120
clone121-125
clone321-325
clone361-365
clone386-390
clone438-442
clone511-515
clone566-570
clone661-665
clone791-795
clone1251-1255
clone1311-1315
clone1381-1385
clone1516-1520
clone1686-1690
clone1696-1700
clone1701-1705
clone1756-1760
clone2091-2095
clone2186-2190
clone2291-2295
clone2541-2545
clone2706-2710
clone2981-2985

Fig. S3

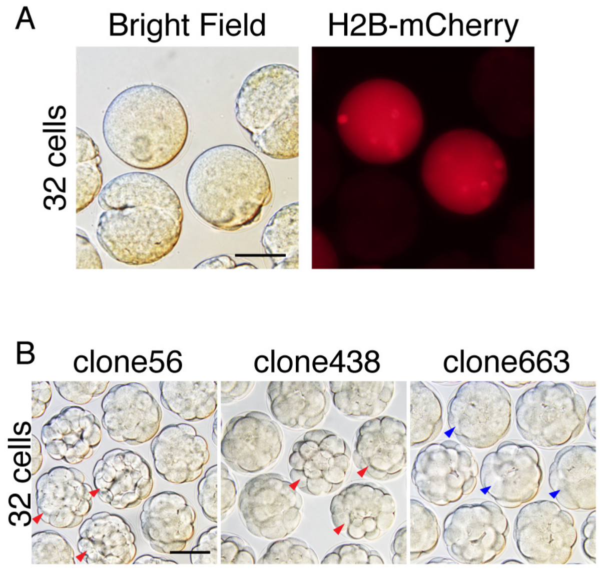

Fig. S5

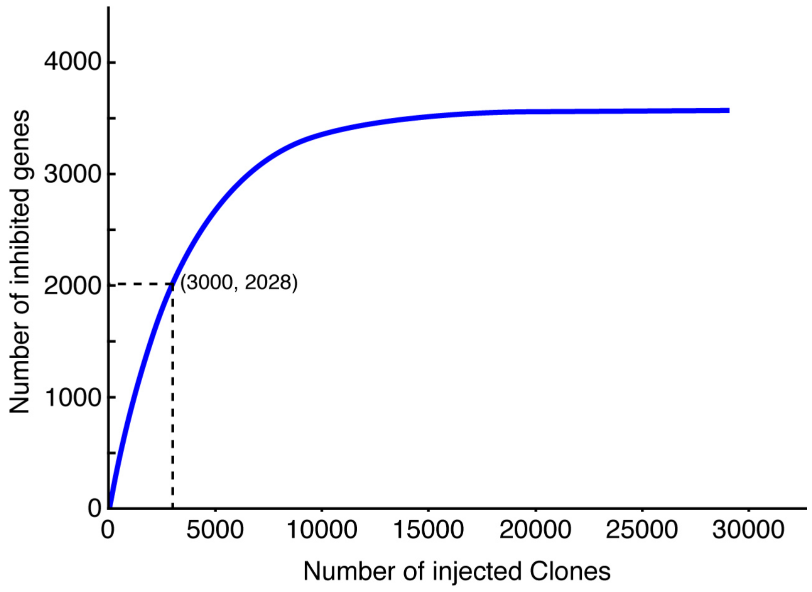

Fig. S4

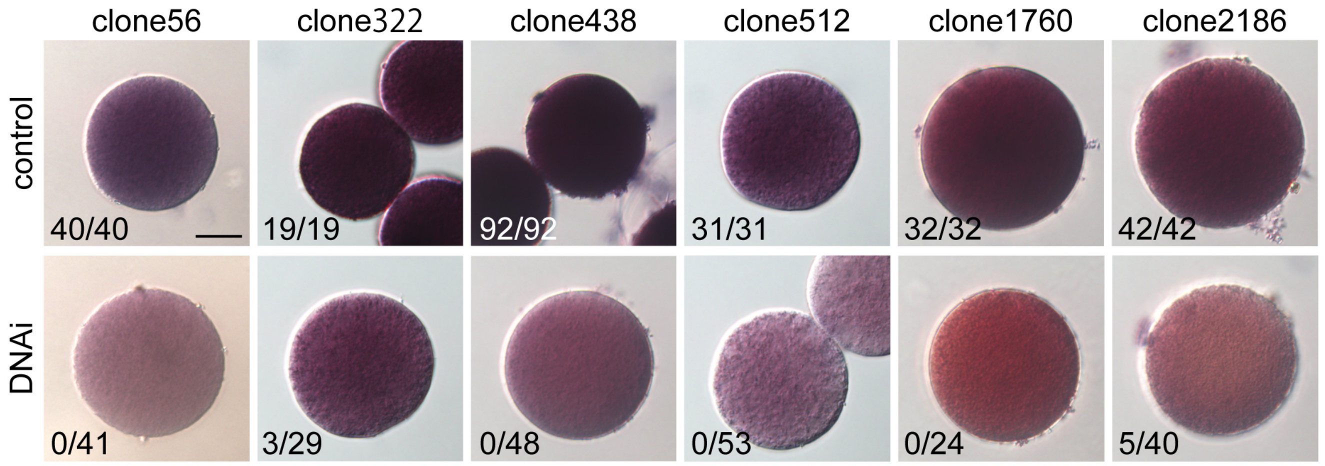

Fig. S6

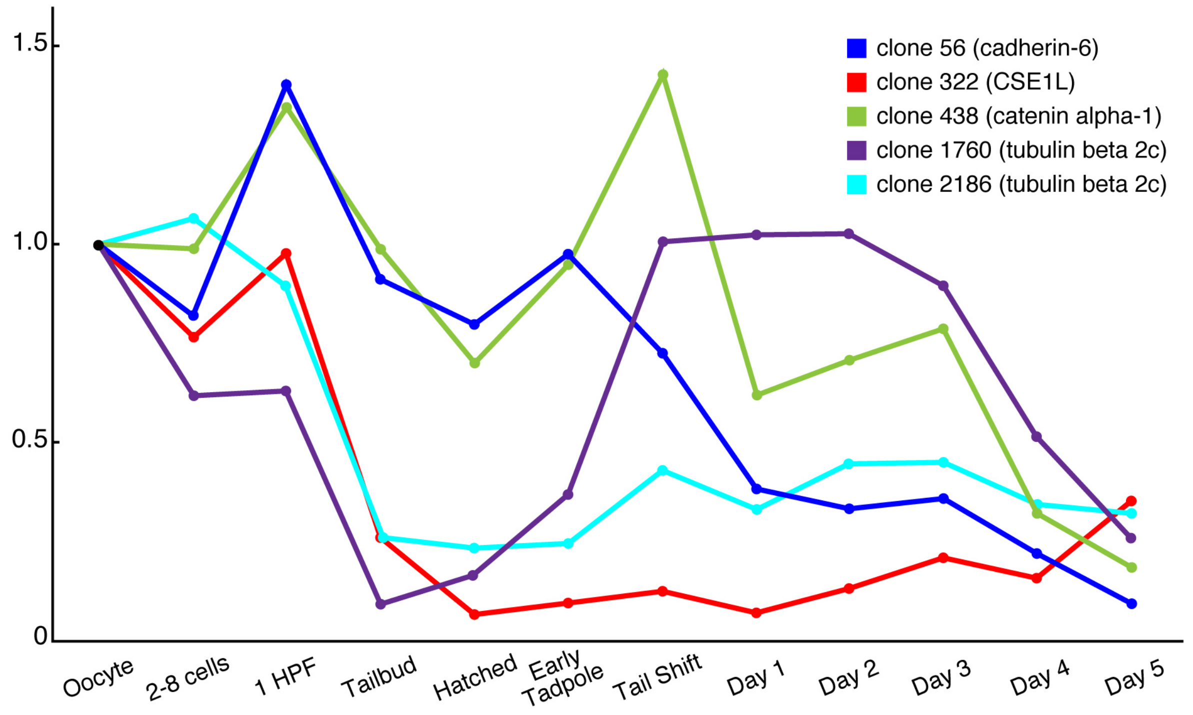

Table S1

clone No.	Protein name	gene ID	Target position	Target length
56	cadherin-6 precursor	GSOIDT00011474001	-193~489	682
438	α-catenin1	GSOIDT00013235001	2368~2776	408
663	rpa-interacting protein a	GSOIDT00013373001	-79~625	704
322	CSE1like	GSOIDT00013374001	1744~2033	289
512	alpha 1b (tubulin)	GSOIDT00000916001	-72~68	140
1760	tubulin β 2c	GSOIDT00006170001	842~1194	352
2186	beta 2c (tubulin)	GSOIDT00002011001	601~844	243

Table S2

clone No.	Protein name	Targeting position	Targeting position
		Ratio of malformation	Ratio of malformation
56	cadherin-6 precursor	-156~161	217~473
		104/106	0/77
438	α-catenin1	2368~2487	2510~2776
		96/97	98/118
663	rpa-interacting protein a	25~213	358~625
		56/71	87/90
322	CSE1like	1744~1845	1890~2007
		13/50	57/73
512	alpha 1b (tubulin)	10~196	208~362
		17/75	12/65
1760	tubulin β 2c	912~1032	1034~1194
		84/93	50/64
2186	beta 2c (tubulin)	601~645	659~844
		66/70	105/106
